# Challenges in multinational rare disease clinical studies during COVID-19: regulatory assessment of cipaglucosidase alfa plus miglustat in adults with late-onset Pompe disease

**DOI:** 10.1007/s00415-024-12843-x

**Published:** 2025-01-07

**Authors:** Benedikt Schoser, Shahram Attarian, Ryan Graham, Fred Holdbrook, Mitchell Goldman, Jordi Díaz-Manera, The ATB200-03 study group

**Affiliations:** 1 Friedrich-Baur-Institute, Department of Neurology, LMU University Clinic, Munich, Germany; 2 Reference Center for Neuromuscular Diseases and ALS, La Timone University Hospital, Aix-Marseille University, Marseille, France; 3 Amicus Therapeutics UK Ltd, Marlow, UK; 4 Amicus Therapeutics, Inc., Princeton, NJ USA; 5 John Walton Muscular Dystrophy Research Centre, Newcastle University, Newcastle-upon-Tyne, UK; 6 Neuromuscular Disorders Unit, Neurology Department, Hospital de La Santa Creu i Sant Pau, Barcelona, Spain; 7 Centro de Investigación Biomédica en Red de Enfermedades Raras (CIBERER), Madrid, Spain

**Keywords:** Glycogen storage disease type II, Alpha glucosidases, Myozyme, Lysosomal storage diseases, Data analyses, Actual time points of assessments

## Abstract

PROPEL (ATB200-03; NCT03729362) compared the efficacy and safety of cipaglucosidase alfa plus miglustat (cipa + mig), a two-component therapy for late-onset Pompe disease (LOPD), versus alglucosidase alfa plus placebo (alg + pbo). The primary endpoint was change in 6-min walk distance (6MWD) from baseline to week 52. During PROPEL, COVID-19 interrupted some planned study visits and assessment windows, leading to delayed visits, make-up assessments for patients who missed ≥ 3 successive infusions before planned assessments at weeks 38 and 52, and some advanced visits (end-of-study/early-termination visits). These were remapped to the respective planned visits. To evaluate if remapping may have overestimated treatment effects, we conducted post hoc analyses using a mixed-effect model for repeated measures based on actual time points of assessments. In this post hoc analysis, estimated mean treatment difference between cipa + mig and alg + pbo for change from baseline to week 52 in 6MWD was 11.7 m (95% confidence interval [CI] − 1.0 to 24.4; *p* = 0.072). In the original published analyses, between-group difference using last observation carried forward was 13.6 m (95% CI − 2.8 to 29.9; *p* = 0.071 [*p* value from separate non-parametric analysis of covariance]). Both statistical analysis approaches led to similar results and consistent conclusions, confirming the efficacy of cipa + mig for adults with LOPD. NCT03729362; trial start date: December 4, 2018.

Trial registration number

**Supplementary Information:**

The online version contains supplementary material available at 10.1007/s00415-024-12843-x.

## Introduction

Pompe disease is a rare, inherited, multisystemic and progressive lysosomal disorder caused by pathogenic variants in the acid α-glucosidase (*GAA*) gene that lead to deficiency of GAA enzyme activity [1, 2]. Accumulation of lysosomal glycogen in muscle causes dysregulated autophagic build-up, resulting in irreversible damage and substitution of muscle tissue by fibrotic and fatty tissue in skeletal, cardiac, and respiratory muscles [1, 3]. Pompe disease is classified into infantile onset and late onset; patients with late-onset Pompe disease (LOPD) may develop symptoms at any age, typically over the age of 1 year [1, 4]. The standard-of-care treatment for symptomatic patients with LOPD is enzyme replacement therapy (ERT) with recombinant human GAA (rhGAA) [5]. Cipaglucosidase alfa plus miglustat (cipa + mig) is a two-component therapy for LOPD that has recently been approved in Europe, the UK, and the USA [6–8].

The pivotal phase III PROPEL study (ATB200-03; NCT03729362) compared the efficacy and safety of cipa + mig versus alglucosidase alfa plus placebo (alg + pbo) in ambulatory adults with LOPD over 52 weeks [9]. The prespecified primary efficacy endpoint was the change in 6-min walk distance (6MWD) in meters from baseline to week 52 to compare cipa + mig against alg + pbo for superiority in the intent-to-treat (ITT) population with observed values (ITT-OBS). The emergence of the COVID-19 pandemic during the study led to some interruptions in the planned study visit and assessment windows, including delayed visits due to COVID-19-related restrictions imposed by various local authorities, make-up assessments for patients who missed three or more successive infusions just before planned study assessments at week 38 and week 52, and some advanced visits (end-of-study/early-termination visits). In the prespecified analysis, the primary efficacy endpoint was planned to be analyzed using a standard mixed-effect model for repeated measures (MMRM). For this analysis, one of the independent variables was the fixed categorical effect of visit, taken as the planned time point of assessment; that is, delayed and advanced visits were remapped to the respective planned study visits, and the make-up assessments at week 52 were used as the week 52 results [9]. However, as the 6MWD data were not normally distributed, an analysis based on the non-parametric randomization-based analysis of covariance (non-parametric ANCOVA) with a last-observation-carried-forward (LOCF) imputation scheme was implemented as specified in the study protocol [9], which replaced missing values with the previous non-missing post-baseline results. Secondary endpoints were analyzed using a parametric ANCOVA model [9].

When the data were reviewed by European regulatory authorities, the Committee for Medicinal Products for Human Use (CHMP) expressed some concerns about delays between planned and actual study visits [10]. The CHMP reviewers felt that remapping of delayed visits may potentially lead to bias in the estimated treatment difference for the primary endpoint, 6MWD. The reviewers also considered the use of the LOCF imputation in the ANCOVA-based analysis for 6MWD to be a non-conservative approach for dealing with missing values that could potentially lead to overestimation of the treatment effect in both groups, particularly for a condition that is expected to lead to deterioration over time, such as LOPD [10]. Therefore, the CHMP requested a post hoc analysis of the primary and key secondary endpoints using an alternative MMRM model based on the actual time points of assessments during the study and without imputation at week 52 [10]. Here, we present the results of these post hoc analyses and assess their consistency with the results of the published analyses.

## Methods

### Study design, procedures, outcomes, and assessments

The PROPEL study, ATB200-03 (NCT03729362), was an international, randomized, double-blind, parallel-group phase III study. The study design, inclusion and exclusion criteria, study procedures, and outcomes have been published previously [9]. Patients in PROPEL had either received alglucosidase alfa at the recommended dose of 20 mg/kg every 2 weeks for at least 2 years (ERT experienced) or had not received previous treatment with alglucosidase alfa or any investigational ERT (ERT naïve). Among the 125 patients enrolled, one ERT-naïve patient in the alg + pbo group was deemed by the principal investigator as likely to have deliberately underperformed at baseline; this outlier patient was excluded from the efficacy analyses [9] as well as the post hoc analysis presented here.

During PROPEL, the COVID-19 pandemic emerged and impeded the conduct of site visits and laboratory testing due to quarantines, travel restrictions, and risk of infection. Patients who missed infusions near the scheduled assessments on week 26, 38, or 52 were to postpone the assessments and receive a sufficient number of “catch-up” infusions before undergoing the study assessments. Every instance of a missed infusion was to be discussed with the medical monitor of the study. If a patient missed five or more consecutive infusions for any reason, including COVID-19, they were withdrawn from the study. If a visit was missed due to COVID-19-related quarantines, travel restrictions, and risk of infection, it was recorded as such. To maintain regular infusions, home infusions were allowed if possible for eligible patients.

The primary efficacy endpoint of the PROPEL study was the change from baseline to week 52 in 6MWD. The first key secondary endpoint was the change from baseline to week 52 in sitting % predicted forced vital capacity (FVC). Other key secondary endpoints included the change from baseline to week 52 in manual muscle test (MMT) score for lower extremities (sum of hip and knee scores), total score for the Patient-Reported Outcomes Measurement Information System (PROMIS) Physical Function and Fatigue measures, and total Gait, Stairs, Gowers’ Maneuver, Chair (GSGC) score. These key secondary endpoints were tested in a hierarchical testing order (order as listed above). Another key secondary endpoint in the original analysis was the change from baseline to week 26 in 6MWD (after MMT lower extremities score in the testing hierarchy). However, this endpoint was not requested for re-analysis in the post hoc analysis.

### Data analysis

#### Original (published) analyses

The original prespecified analysis methodology has been described [9]. In brief, the primary efficacy endpoint (change in 6MWD from baseline to week 52) was planned to be analyzed with an MMRM model to compare for superiority of cipa + mig to alg + pbo in the ITT-OBS population, using all available observed data without imputation for missing post-baseline data. The response variable in the original MMRM analysis was the change from baseline to all post-baseline visits. Independent variables were the fixed, categorical effects of treatment, time (planned study visit number), treatment-by-visit interaction, previous ERT status (ERT experienced or ERT naïve), and gender, as well as the fixed, continuous covariates of baseline 6MWD, baseline age, baseline weight, and baseline height.

In the original analysis, results of delayed visits were remapped to planned study visits. An unstructured covariance structure was used to model the within-patient variability. The criteria for assessing the normality of the MMRM model were prespecified; as normality assumptions were violated for the 6MWD data, a prespecified non-parametric randomization-based ANCOVA [11, 12] adjusting for treatment, previous ERT status, gender, baseline endpoint value, age, weight, and height was performed on the ITT population, using the LOCF imputation (ITT-LOCF population) [9].

The key secondary endpoints were analyzed using a parametric ANCOVA model, adjusting for treatment, previous ERT status, gender, baseline endpoint value, age, weight, and height, on the ITT-LOCF population and compared between treatment groups. To control the overall alpha level, a hierarchical testing procedure was planned with ordering of the secondary endpoints to be tested sequentially after the primary efficacy endpoint was tested to be statistically significant.

#### Post hoc analyses

The new post hoc analysis employed an MMRM model based on the ITT-OBS population, using the actual time point of the assessments and without imputation at week 52 [10]. The new MMRM model included the fixed, categorical effects of treatment, previous ERT status, and gender, as well as the fixed, continuous covariates of time of assessment (days), baseline 6MWD, age, weight, height, and the treatment-by-time interaction. A random intercept of patient was also included in the model, and a compound symmetry covariance structure was applied. This model was used to analyze the primary and key secondary endpoints. Data were analyzed for the overall ITT-OBS population and in subgroups of patients based on previous ERT status.

## Results

### Protocol deviations

Between December 3, 2018, and November 26, 2019, 130 patients were screened for eligibility for PROPEL, with 125 patients enrolled and randomly assigned to receive cipa + mig (*n* = 85) or alg + pbo (*n* = 40). Two patients in the alg + pbo group did not receive any treatment and were excluded from the ITT population [9]. In total, 66 (53.7%) patients had protocol deviations due to the COVID-19 pandemic, 47 (55.3%) in the cipa + mig group and 19 (50.0%) in the alg + pbo group [9]; 17 patients had a delayed visit of at least 28 days after the target day (13 in the cipa + mig group and four in the alg + pbo group), including eight patients with a delay of at least 42 days (all in the cipa + mig group).

### 6MWD

**Fig. 1:**
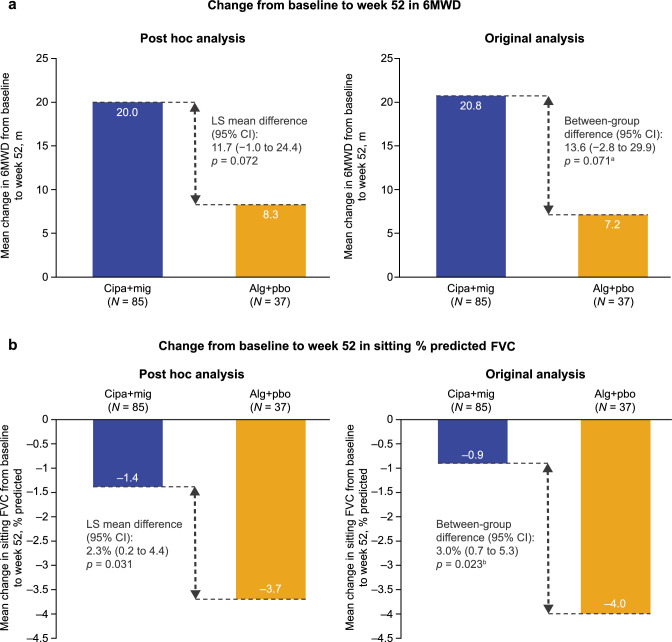
Change from baseline to week 52 in **a** 6MWD and **b** sitting % predicted FVC (ITT population excluding outlier). In the original analysis, values for change from baseline to week 52 were based on LOCF data. *p* values were nominal two sided. The post hoc analysis used MMRM with actual time points of assessments in the ITT-OBS population. The number of patients in the post hoc analysis depended on the number of patients with non-missing values for the analysis. In all analyses, results exclude one patient judged likely to have deliberately underperformed at baseline. ^a^As the 6MWD data were not normally distributed, the *p* value for 6MWD was from non-parametric ANCOVA as prespecified; ^b^For sitting % predicted FVC, the *p* value in the original analysis was from ANCOVA as prespecified. *6MWD* 6-min walk distance, *alg* + *pbo* alglucosidase alfa plus placebo, *ANCOVA* analysis of covariance, *CI* confidence interval, *cipa* + *mig* cipaglucosidase alfa plus miglustat, *FVC* forced vital capacity, *ITT* intent to treat, *ITT-OBS* ITT population with observed values, *LOCF* last observation carried forward, *LS* least squares, *MMRM* mixed-effect model for repeated measures

In the original analyses in the overall population, mean (standard error [SE]) change from baseline to week 52 in 6MWD was 20.8 m (4.6) in the cipa + mig group versus 7.2 m (6.6) in the alg + pbo group using LOCF, with a between-group difference of 13.6 m (95% confidence interval [CI] − 2.8 to 29.9; Fig. 1a) [9]. As the 6MWD data were not normally distributed, the prespecified non-parametric ANCOVA analysis was used to compare the treatment groups (*p* = 0.071). The least-squares (LS) mean difference in change from baseline to week 52 in 6MWD between the two treatments (using LOCF) was 13.7 m (95% CI − 1.2 to 28.5) [9].

In the post hoc analysis, based on the actual time point of assessments, the estimated mean change from baseline to week 52 (LS mean) in 6MWD was 20.0 m (95% CI 13.1 to 26.9; standard deviation [SD] 3.5) in the cipa + mig group (baseline mean 357.9 m; SD 111.8; median 359.9; *n* = 85) and 8.3 m (95% CI − 2.2 to 18.8; SD 5.3) in the alg + pbo group (baseline mean 351.0 m; SD 121.3; median 365.5; *n* = 37; Fig. 1a). The estimated mean treatment difference was 11.7 m (95% CI − 1.0 to 24.4; SE 6.4), with a two-sided *p* value of 0.072 (Fig. 1a). As in the original analysis, statistical superiority (*p* < 0.05) was not demonstrated for cipa + mig in the post hoc analysis for the ITT-OBS population, which included both ERT-experienced and ERT-naïve patients.

### Sitting % predicted FVC

In the original analyses, the ANCOVA model for the first key secondary endpoint of sitting % predicted FVC satisfied the normality assumption. Mean (SE) change from baseline to week 52 was − 0.9% (0.7) in the cipa + mig group and − 4.0% (0.8) in the alg + pbo group, with a between-group difference of 3.0% (95% CI 0.7 to 5.3; *p* = 0.023 [*p* value from prespecified ANCOVA]; Fig. 1b) [9]. The LS mean difference in change from baseline to week 52 in sitting % predicted FVC between the two treatments was 2.7% (95% CI 0.4 to 5.0) [9].

In the post hoc analysis, the estimated mean change from baseline to week 52 in sitting % predicted FVC was − 1.4 (95% CI − 2.5 to − 0.3; SD 0.6) in the cipa + mig group (baseline mean 70.7%; SD 19.6; median 70.0; *n* = 85) and − 3.7 (95% CI − 5.4 to − 2.0; SD 0.9) in the alg + pbo group (baseline mean 69.7%; SD 21.5; median 71.0; *n* = 37) of the ITT OBS-population. The estimated mean treatment difference was 2.3% (95% CI 0.2 to 4.4; SE 1.1), with a two-sided *p* value of 0.031 (Fig. 1b). As in the original analysis, cipa + mig was nominally significantly superior to alg + pbo for the ITT-OBS population.

### Other key secondary endpoints

**Fig. 2:**
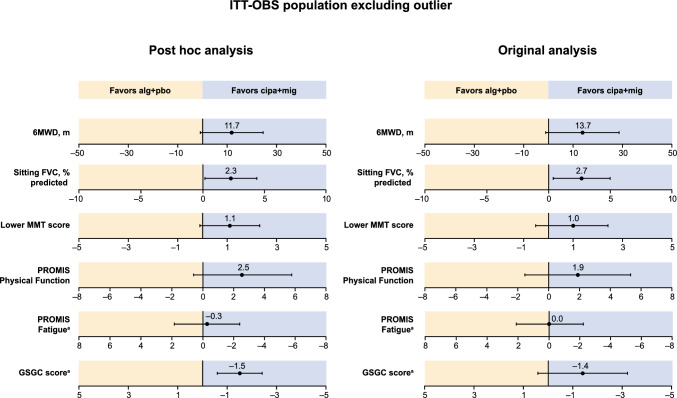
LS mean treatment difference for change from baseline to week 52 for the primary and key secondary endpoints (ITT population excluding outlier). Data shown are LS mean treatment differences. Error bars indicate 95% CI. In the original analysis, change from baseline to week 52 are LOCF means, and LS mean differences were based on ANCOVA models adjusting for baseline covariates except for 6MWD, which used non-parametric ANCOVA and differed slightly from the mean differences shown in Fig. 1 for 6MWD and sitting % predicted FVC. The post hoc analysis used MMRM with actual time points of assessments in the ITT-OBS population. ^a^For these endpoints, a negative change from baseline indicates an improvement, and axis values have been reversed. *6MWD* 6-min walk distance, *alg* + *pbo* alglucosidase alfa plus placebo, *ANCOVA* analysis of covariance, *CI* confidence interval, *cipa* + *mig* cipaglucosidase alfa + miglustat, *FVC* forced vital capacity, *GSGC* Gait, Stairs, Gowers’ Maneuver, Chair, *ITT* intent to treat, *ITT-OBS* ITT population with observed values, *LOCF* last observation carried forward, *LS* least squares, *MMRM* mixed-effect model for repeated measures, *MMT* manual muscle test, *PROMIS* Patient-Reported Outcomes Measurement Information System

The results of the post hoc MMRM analysis for the other key secondary endpoints (lower MMT score, PROMIS Physical Function score, PROMIS Fatigue score, and GSGC total score) in the ITT-OBS population were also similar to those of the original analyses. All key secondary endpoints numerically favored cipa + mig versus alg + pbo in the original and post hoc analyses, except for PROMIS Fatigue score, which showed no difference between treatment groups in the original analyses or the post hoc analysis (Fig. 2 and Supplementary Table S1). Treatment differences were not statistically significant except for GSGC score in the post hoc analysis (two-sided *p* value of 0.001; Supplementary Table S1).

### Subgroup analysis by previous ERT status

**Fig. 3:**
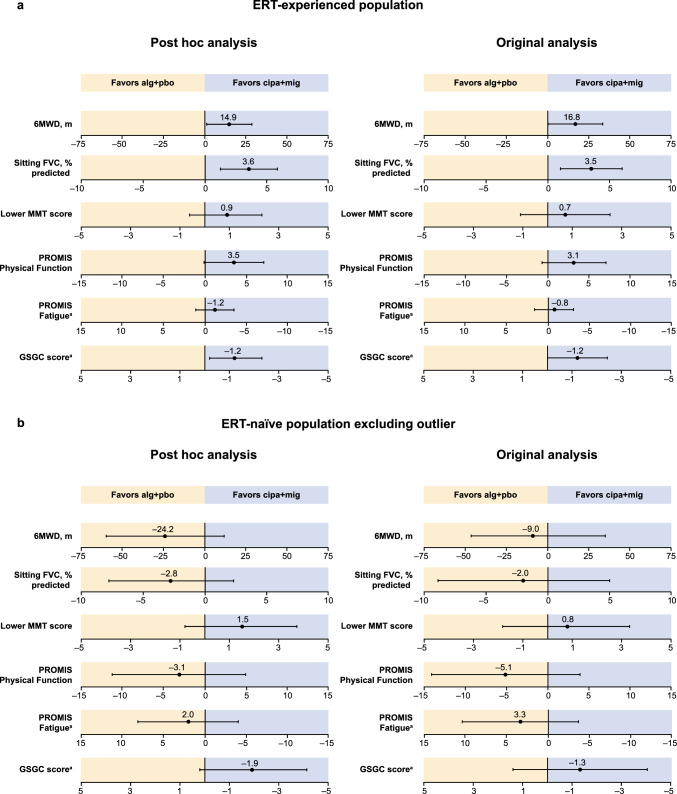
LS mean treatment difference for change from baseline to week 52 for the primary and key secondary endpoints in **a** ERT-experienced and **b** ERT-naïve patients. Data shown are LS mean treatment difference. Error bars indicate 95% CI. In the original analysis, change from baseline to week 52 were LOCF means. LS mean differences were based on analysis models adjusting for baseline covariates. The post hoc analysis used MMRM with actual time points of assessments in the ITT-OBS population. ^a^For these endpoints, a negative change from baseline indicates an improvement, and axis values have been reversed. *6MWD* 6-min walk distance, *alg* + *pbo* alglucosidase alfa plus placebo, *CI* confidence interval, *cipa* + *mig* cipaglucosidase alfa plus miglustat, *ERT* enzyme replacement therapy, *FVC* forced vital capacity, *GSGC* Gait, Stairs, Gowers’ Maneuver, Chair, *ITT-OBS* intent-to-treat population with observed values, *LOCF* last observation carried forward, *LS* least squares, *MMRM* mixed-effect model for repeated measures, *MMT* manual muscle test, *PROMIS* Patient-Reported Outcomes Measurement Information System

Similar to the post hoc analysis in the overall population, analysis of the primary and key secondary endpoints in subgroups of ERT-experienced and ERT-naïve patients showed the same trends as the original analyses in both subgroups (Fig. 3). In ERT-experienced patients, nominal significance in favor of cipa + mig versus alg + pbo was reached for the primary endpoint of change from baseline to week 52 in 6MWD and for the key secondary endpoint of change from baseline to week 52 in sitting % predicted FVC in both the original analysis [9] and the post hoc analysis (Supplementary Table S2). In ERT-naïve patients, some endpoints favored cipa + mig while others favored alg + pbo in the original analyses, and the same pattern was seen in the post hoc analysis (Fig. 3b and Supplementary Table S3).

## Discussion

In the original, protocol-prespecified analysis of the PROPEL study, the primary endpoint (the difference in change from baseline to week 52 in 6MWD between cipa + mig and alg + pbo) was analyzed using an MMRM model based on the ITT-OBS population, and because the 6MWD data were not normally distributed, a prespecified non-parametric randomization-based ANCOVA with the LOCF imputation was applied [9]. Cipa + mig was recently granted approval by the US Food and Drug Administration for the treatment of adult patients with LOPD weighing ≥ 40 kg and who are not improving on their current ERT [8]. However, it is not unusual for regulatory authorities to have different requirements in terms of trial design, preferred endpoints, and data analysis when evaluating new drug applications [13–15], thus it is important to consider relevant requirements when designing clinical trials and to accommodate post hoc analyses during regulatory review if requested.

The PROPEL study enrolled patients between December 2018 and November 2019 [9]. From March 2020, the COVID-19 pandemic and associated restrictions had a global impact on the conduct of clinical trials [16, 17]. In PROPEL, not all study visits and assessments could be performed within the prespecified visit window (± 3 days for assessment visits at weeks 12, 26, 38, and 52 or end of treatment). Therefore, the European Medicines Agency (EMA) requested post hoc analyses to evaluate whether the remapping of delayed (or advanced) visits may have led to overestimation of the treatment effect of cipa + mig versus alg + pbo. MMRM analysis using the actual time points of the assessments was expected to result in a slightly different estimate of the treatment difference compared with MMRM analysis based on the planned fixed visit numbers, especially when the assessments were not performed at the planned visits [10].

For PROPEL, both the original, published statistical analyses and the EMA-requested post hoc analyses led to similar results in the overall population and in subgroups of ERT-experienced and ERT-naïve patients [9, 10]. In both analyses, in the overall population, cipa + mig led to improvements in 6MWD and stabilization in sitting % predicted FVC compared with study baseline that were considered to be clinically relevant [10, 18]. However, statistical superiority versus alg + pbo for improvements from baseline in 6MWD was not demonstrated in the overall population; therefore, based on the testing hierarchy, the improved outcome for sitting % predicted FVC was considered only nominally significant. Subgroup analysis based on ERT status before study entry showed that, for ERT-experienced patients, switching from alg + pbo to cipa + mig led to improved performance in 6MWD and sitting % predicted FVC compared with patients remaining on alg + pbo, the current standard of care [18]. For ERT-naïve patients, both cipa + mig and alg + pbo led to improvements compared with baseline in 6MWD, but the treatment benefit with cipa + mig was not superior to that with alg + pbo in either analysis.

Challenges with conducting clinical trials during the COVID-19 pandemic included a decline in patient enrollment, limited accessibility of clinics for essential visits, reluctance of patients to visit clinics, risk of acquiring the infection for patients and study staff, higher study dropout rates, and deviations from the study timelines that may affect data integrity because of delayed or missed assessment and monitoring [19]. During PROPEL, the pandemic led to some restrictions in accessibility of clinics for planned assessment visits and reluctance of some patients to visit clinics, highlighting a need to develop alternative measures that could be performed remotely [19], particularly as patients with Pompe disease were considered to be at increased risk of severe COVID-19 disease during the pandemic [20].

### Limitations

The small sample size limited interpretation of the results for the ERT-naïve cohort; other limitations of this post hoc analysis are the same as for the original analyses [9].

## Conclusions

Although the COVID-19 pandemic led to delayed study visits for some patients participating in the PROPEL study, the two statistical analyses demonstrated the efficacy of cipa + mig for adult patients with LOPD, as reflected in the label granted by the EMA. The similarity of the results of the post hoc analysis to those of the original planned analysis supports the robustness of the original analysis.

## Supplementary Information

Below is the link to the electronic supplementary material.

Supplementary file1 (PDF 168 KB)

## Data Availability

Data sharing proposals and requests will be reviewed on a case-by-case basis. Requests for data should be addressed to Nicholas A. Rees at nrees@amicusrx.com. Requests will be reviewed by a medical steering committee.

## Abbreviations

6MWD: 6-Min walk distance
alg+pbo: alglucosidase alfa plus placebo
cipa+mig: cipaglucosidase alfa plus miglustat
ANCOVA: Analysis of covariance
CHMP: Committee for Medicinal Products for Human Use
CI: Confidence interval
EMA: European Medicines Agency
ERT: Enzyme replacement therapy
FVC: Forced vital capacity
GAA: Acid α-glucosidase
GSGC: Gait, Stairs, Gowers’ Maneuver, Chair
ITT: Intent to treat
ITT-OBS: Intent-to-treat population with observed values
LOCF: Last observation carried forward
LOPD: Late-onset Pompe disease
LS: Least squares
MMRM: Mixed-effect model for repeated measures
MMT: Manual muscle test
PROMIS: Patient-Reported Outcomes Measurement Information System
rhGAA: Recombinant human acid α-glucosidase
SD: Standard deviation
SE: Standard error
