# Supplementary material for: Challenges in multinational rare disease clinical studies during COVID-19: regulatory assessment of cipaglucosidase alfa plus miglustat in adults with late-onset Pompe disease
Source: J Neurol. 2025 Jan 7;272(1):103. doi: 10.1007/s00415-024-12843-x (PMC11706903; doi:10.1007/s00415-024-12843-x)
Supplement: Supplementary file 1 — Supplementary file1 (PDF 168 KB) [file 415_2024_12843_MOESM1_ESM.pdf]

Supplementary material

Challenges in multinational rare disease clinical studies during COVID-19: regulatory assessment of cipaglucosidase alfa plus miglustat in adults with late-onset Pompe disease

Benedikt Schoser,[1] Shahram Attarian,[2] Ryan Graham,[3] Fred Holdbrook,[4] Mitchell Goldman,[4] Jordi Díaz-Manera,[5,6,7] on behalf of the ATB200-07 study group

[1]Friedrich-Baur-Institute, Department of Neurology, LMU University Clinic, Munich, Germany; [2]Reference Center for Neuromuscular Diseases and ALS, La Timone University Hospital, Aix-Marseille University, Marseille, France; [3]Amicus Therapeutics UK LTD, Marlow, UK; [4]Amicus Therapeutics, Inc., Princeton, NJ, USA; [5]John Walton Muscular Dystrophy Research Centre, Newcastle University, Newcastle upon Tyne, UK; [6]Neuromuscular Disorders Unit, Neurology Department, Hospital de la Santa Creu i Sant Pau, Barcelona, Spain; [7]Centro de Investigación Biomédica en Red de Enfermedades Raras (CIBERER), Madrid, Spain.

Journal title: *Journal of Neurology*

Corresponding author: Prof Dr med Benedikt Schoser; Friedrich-Baur-Institute, Department of Neurology, LMU University Clinic, Munich, Germany; benedikt.schoser@med.uni-muenchen.de

Supplementary Table S1. Post hoc analysis of primary and key secondary endpoints (ITT-OBS population excluding outlier)

	LS mean change (95% CI) from baseline to week 52			
	**Cipa+mig**	**Alg+pbo**	**LS mean treatment difference (95% CI)**	**Two-sided *p* value**
**6MWD, m**	20.0 (13.1 to 26.9)	8.3 (−2.2 to 18.8)	11.7 (−1.0 to 24.4)	0.072
**Sitting % predicted FVC**	−1.4 (−2.5 to −0.3)	−3.7 (−5.4 to −2.0)	2.3 (0.2 to 4.4)	0.031
**Lower MMT score**	1.7 (1.1 to 2.4)	0.7 (−0.4 to 1.7)	1.1 (−0.1 to 2.3)	0.077
**PROMIS Physical Function score**	2.2 (0.5 to 3.9)	−0.3 (−2.9 to 2.3)	2.5 (−0.6 to 5.7)	0.113
**PROMIS Fatigue score[a]**	−2.0 (−3.2 to −0.9)	−1.7 (−3.4 to 0.0)	−0.3 (−2.4 to 1.8)	0.748
**GSGC total score[a]**	−0.7 (−1.2 to −0.2)	0.8 (0.0 to 1.5)	−1.5 (−2.4 to −0.6)	0.001

[a]For these endpoints, a negative change from baseline indicates an improvement. *6MWD* 6-minute walk distance; *alg+pbo* alglucosidase alfa plus placebo; *CI* confidence interval; *cipa+mig* cipaglucosidase alfa plus miglustat; *FVC* forced vital capacity; *GSGC* Gait, Stairs, Gowers' Maneuver, Chair; *ITT-OBS* intent-to-treat population with observed values; *LS* least squares; *M* meter; *MMT* manual muscle test; *PROMIS* Patient-Reported Outcomes Measurement Information System

Supplementary Table S2. Post hoc analysis of primary and key secondary endpoints in ERT-experienced patients (ITT-OBS population)

	LS mean change (95% CI) from baseline to week 52			
	**Cipa+mig**	**Alg+pbo**	**LS mean treatment difference (95% CI)**	**Two-sided *p* value**
**6MWD, m**	15.8 (8.3 to 23.4)	0.9 (−10.2 to 12.1)	14.9 (1.2 to 28.6)	0.033
**Sitting % predicted FVC**	−0.2 (−1.5 to 1.1)	−3.8 (−5.7 to −1.9)	3.6 (1.3 to 5.9)	0.002
**Lower MMT score**	1.8 (1.0 to 2.6)	0.9 (−0.3 to 2.1)	0.9 (−0.6 to 2.3)	0.255
**PROMIS Physical Function score**	2.0 (−0.0 to 4.0)	−1.6 (−4.5 to 1.4)	3.5 (−0.1 to 7.1)	0.056
**PROMIS Fatigue score[a]**	−1.9 (−3.2 to −0.6)	−0.7 (−2.6 to 1.2)	−1.2 (−3.5 to 1.1)	0.310
**GSGC total score[a]**	−0.7 (−1.3 to −0.1)	0.5 (−0.4 to 1.4)	−1.2 (−2.2 to −0.1)	0.028

[a]For these endpoints, a negative change from baseline indicates an improvement. *6MWD* 6-minute walk distance; *alg+pbo* alglucosidase alfa plus placebo; *CI* confidence interval; *cipa+mig* cipaglucosidase alfa plus miglustat; *ERT* enzyme replacement therapy; *FVC* forced vital capacity; *GSGC* Gait, Stairs, Gowers' Maneuver, Chair; *ITT-OBS* intent-to-treat population with observed values; *LS* least squares; *M* meter; *MMT* manual muscle test; *PROMIS* Patient-Reported Outcomes Measurement Information System

Supplementary Table S3. Post hoc analysis of primary and key secondary endpoints in ERT-naïve patients (ITT-OBS population excluding outlier)

	LS mean change (95% CI) from baseline to week 52			
	**Cipa+mig**	**Alg+pbo**	**LS mean treatment difference (95% CI)**	**Two-sided *p* value**
**6MWD, m**	28.5 (12.4 to 44.7)	52.7 (23.2 to 82.3)	−24.2 (−60.0 to 11.7)	0.178
**Sitting % predicted FVC**	−5.2 (−7.5 to −2.9)	−2.4 (−6.7 to 1.8)	−2.8 (−7.8 to 2.3)	0.271
**Lower MMT score**	1.4 (0.4 to 2.5)	−0.0 (−1.9 to 1.9)	1.5 (−0.8 to 3.7)	0.198
**PROMIS Physical Function score**	2.6 (−1.0 to 6.3)	5.8 (−0.9 to 12.4)	−3.1 (−11.2 to 4.9)	0.431
**PROMIS Fatigue score[a]**	−3.0 (−5.7 to −0.2)	−4.9 (−9.9 to 0.0)	2.0 (−4.1 to 8.0)	0.506
**GSGC total score[a]**	−0.6 (−1.6 to 0.4)	1.3 (−0.4 to 3.1)	−1.9 (−4.1 to 0.2)	0.074

[a]For these endpoints, a negative change from baseline indicates an improvement. *6MWD* 6-minute walk distance; *alg+pbo* alglucosidase alfa plus placebo; *CI* confidence interval; *cipa+mig* cipaglucosidase alfa plus miglustat; *ERT* enzyme replacement therapy; *FVC* forced vital capacity; *GSGC* Gait, Stairs, Gowers' Maneuver, Chair; *ITT-OBS* intent-to-treat population with observed values; *LS* least squares; *M* meter; *MMT* manual muscle test; *PROMIS* Patient-Reported Outcomes Measurement Information System
